# Steroid-resistant nephrotic syndrome associated with certain *SGPL1* variants in a family: Case report and literature review

**DOI:** 10.3389/fped.2023.1079758

**Published:** 2023-02-16

**Authors:** Siying Yang, Yonghua He, Jianhua Zhou, Huiqing Yuan, Liru Qiu

**Affiliations:** The Nephrology Division of Department of Pediatrics, Tongji Hospital, Tongji Medical College, Huazhong University of Science and Technology, Wuhan, China

**Keywords:** steroid-resistant nephrotic syndrome, proteinuria, SGPL1 gene mutation, clinical phenotype, genotype

## Abstract

**Objectives:**

Steroid-resistant nephrotic syndrome (SRNS) is a clinical syndrome characterized by the lack of response to standard steroid therapy, usually progressing to end-stage renal disease. We reported two cases of female identical twins with SRNS caused by *SGPL1* variants in one family, reviewed the relevant literature, and summarized their clinical phenotypes, pathological types, and genotypic characteristics.

**Methods:**

Two cases of nephrotic syndrome caused by *SGPL1* variants were admitted to Tongji Hospital, affiliated with Tongji Medical College of Huazhong University of Science and Technology. Their clinical data were retrospectively collected, and the peripheral blood genomic DNA was captured and sequenced by whole exome sequencing. Related literature published in PubMed, CNKI, and Wan fang databases was reviewed.

**Results:**

We described two Chinese identical twin girls with isolated SRNS due to compound heterozygous variants in the *SGPL1* (intron4 c.261 + 1G > A and intron12 c.1298 + 6T > C). The patients were followed up for 60.0 months and 53.0 months, respectively, having no extra-renal manifestations. They all died due to renal failure. A total of 31 children with *SGPL1* variants causing nephrotic syndrome (including the reported two cases) were identified through a literature review.

**Conclusions:**

These two female identical twins were the first reported cases of isolated SRNS caused by *SGPL1* variants. Almost all homozygous and compound heterozygous variants of *SGPL1* had extra-renal manifestations, but compound heterozygous variants in the intron of *SGPL1* may have no obvious extra-renal manifestations. Additionally, a negative genetic testing result does not completely rule out genetic SRNS because the Human Gene Mutation Database or ClinVar is constantly being updated.

## Introduction

*SGPL1* (MIM# 603729) encodes sphingosine-1-phosphate lyase (SPL). SPL is an endoplasmic reticulum enzyme involved in sphingolipid catabolism that catalyzes the cleavage of sphingosine-1-phosphate (S1P), leading to its irreversible degradation to ethanolamine phosphate and hexadecenal (1). S1P is a significant bioactive sphingolipid metabolite that regulates various physiological processes and plays a role in the pathogenesis of many diseases, including cancer, atherosclerosis, diabetes mellitus, and osteoporosis (2). It is also a powerful signaling molecule that regulates a wide range of cellular functions, such as cell proliferation, differentiation, and migration, as well as vascular maturation, angiogenesis, and immune function. SPL does this by binding to the five S1P receptors (S1PR1-S1PR5) in the G protein-coupled receptor family (3).

In 2017, Prasad and Lovric et al. found that variants in *SGPL1* can lead to steroid-resistant nephrotic syndrome (SRNS) (NPHS14, OMIM 617575) with an autosomal recessive mode of inheritance. Prasad et al. identified 4 different variants of *SGPL1* in five families in which patients were diagnosed with SRNS, primary adrenal insufficiency, primary hypothyroidism, neurological symptoms and cryptorchidism (4). In the same journal, Lovric and colleagues identified 9 different recessive variants of *SGPL1* in seven families with SRNS. On renal biopsies, focal segmental glomerulosclerosis (FSGS) and diffuse mesangial sclerosis (DMS) were observed. Most patients progressed to end-stage renal disease (ESRD) or died in infancy and early childhood. The patients exhibited a syndromic form of SRNS that presented with additional clinical features. Extra-renal manifestations included adrenal insufficiency, ichthyosis, other endocrine or gonadal defects and neurological deficits including microcephaly, cranial nerve defects and peripheral neuropathy. Approximately half of the patients with the condition were noted to have severe immunodeficiency manifesting as lymphopenia and multiple bacterial infections. Besides, some patients also exhibited failure to thrive and many families reported a history of prior fetal loss (5). NPHS14 is a novel childhood syndrome that features a wide range of presentations, including SRNS, fetal hydrops, primary adrenal insufficiency, rapid or insidious neurological deterioration, immunodeficiency, ichthyosis, and endocrine abnormalities (4–6). Now, this condition is referred to as sphingosine phosphate lyase insufficiency syndrome (SPLIS) (1).

In this paper, two cases of isolated SRNS caused by complex heterozygous variants in the intron of *SGPL1* were reported for the first time. By reviewing the related literature, we found that both homozygous and compound heterozygous variants in *SGPL1* almost always had extra-renal manifestations, but the two patients with compound heterozygous variants in the intron of *SGPL1* in this paper had no obvious extra-renal manifestations during follow-up.

## Materials and methods

### Data collection

We reported two identical twin females with *SGPL1* variants causing nephrotic syndrome (NS) that were diagnosed in May 2016 at Tongji Hospital, Tongji Medical College, Huazhong University of Science and Technology, China. Basic information, laboratory tests, renal pathological features, genetic testing results, and prognosis were collected and analyzed in detail. The Schwartz formula was used to calculate the estimated glomerular filtration rate (eGFR) to evaluate renal function.

### Whole exon sequencing

Five ml of peripheral blood was drawn from the two cases. Genomic DNA was extracted according to the instructions of the kit (MagPure Buffy Coat DNA Midi KF Kit, Guangzhou, China). The genomic DNA was interrupted (segmentation enzyme: shearing enzyme premix reagent) and then screened and purified (Enzymatic, Vahtstm DNA Clean beads) to construct a DNA library of the affected samples. Capture samples were obtained with a gene fragment capture probe (KAPA Hyper Exome), and post-polymerase chain reaction (PCR) reactions were performed. A high-throughput sequencer (Shenzhen UWI MGISEQ-2000) was used for sequencing, and the sequencing type was PE100 + 10. Subsequently, annotation and screening of the suspected variants were performed (BWA software and GATK software; databases: NCBI dbSNP, HapMap, 1000 human genome dataset, and database of 100 healthy Chinese adults). For all identified variants, primers were designed upstream and downstream of their fragments, and the products were sequenced by Sanger using PCR amplification to verify the results of gene chip capture and high-throughput sequencing.

### Literature

PubMed, CNKI, and Wan fang databases were searched for the subject terms “*SGPL1*[Abstract]” and “NPHS14.” Afterward, the Endnote was used to check the duplicate of the retrieved literature. After reading all the relevant literature, we selected the papers based on the following criteria: (a) the papers that included clinical cases; (b) the patients' genetic testing results were identified as *SGPL1* variants; (c) the cases had renal symptoms, such as NS. The excluded criteria were (a) the papers without clinical cases; (b) the papers that summarized cases from other articles without reporting new cases.

## Results

### Case presentation

**Figure 1:**
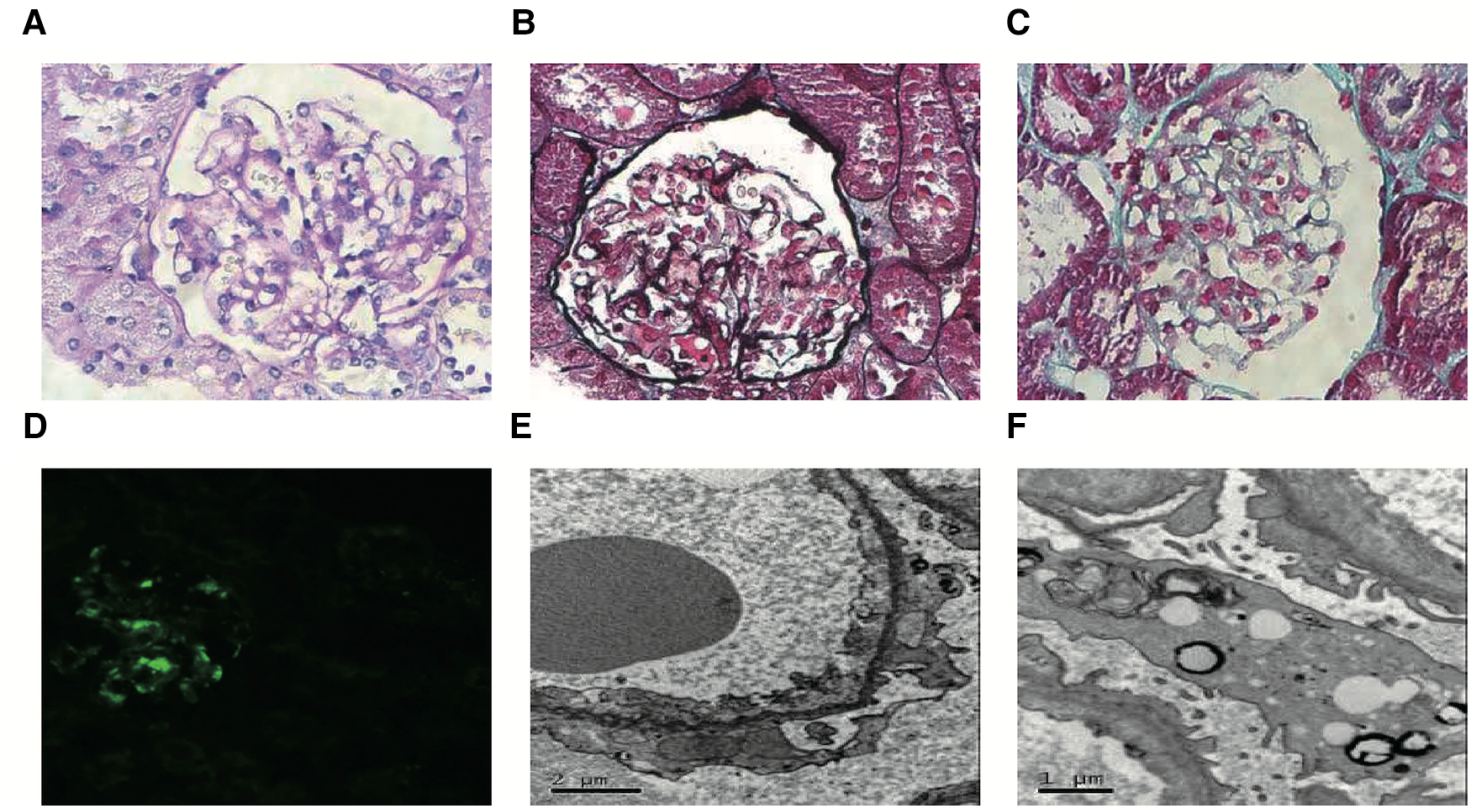
Representative images of renal pathology in case 1. Light microscopy: Most glomerular segmental mesangial cells were mildly hyperplasia, and the mesangial stroma was hyperplasia. There was no obvious lesion in the glomerular basement membrane, and no dense deposits were seen under the endothelial and epithelium. Renal tubular epithelial cells showed degeneration. Atrophy was seen in some segments of renal tubules, and the basement membrane of renal tubules was thickened in some segments. The renal interstitium was widened, and flaky fibrosis was seen. (**A**, PAS, magnification × 400; **B**, PASM, magnification × 400; **C**, Masson, magnification × 400). Immunofluorescence microscopy: IgM (+++) was deposited in the glomerular mesangial region and capillary wall as a mass of immunofluorescence. (**D**, magnification × 200). Transmission electron microscopy: Most segments of the capillary basement membrane had no obvious lesions, and a few segments showed mesangial insertion. The rough endoplasmic reticulum in podocytes expanded, mitochondria swelled, small lipid droplets and lysosomes increased slightly, and foot processes fused widely. The mesangial matrix was mild to moderate hyperplasia, and a small amount of the dense deposits was seen in the mesangial area (**E,F**).

A 5-year-old girl (case 1) was already diagnosed with NS due to nephrotic-range proteinuria in February 2016. Similarly, her identical twin sister (case 2) also developed nephrotic-range proteinuria in February 2016. Both two cases received an adequate oral dose of prednisone (2 mg/kg/days) but did not experience a decrease in urinary protein levels after four weeks. This led to the diagnosis of SRNS, but the condition did not improve even after the addition of cyclosporine. Three months later, they were referred to our hospital for an abnormal urine test and swelling in the lower limbs. Following our recommendations, case 1 underwent whole exome sequencing (WES) and renal puncture biopsy. IgM nephropathy with glomerulosclerosis and interstitial tubular lesions was diagnosed by renal biopsy (Figure 1). According to the genetic testing report, neither the subject samples (including case 2) nor the parental samples contained any single nucleotide variants (SNVs) and insertions and deletions (INDELs) associated with their phenotypes. During the initial 2-year follow-up, both patients presented with SRNS and continued to have nephrotic range proteinuria after progressive multitarget therapy (tacrolimus, mycophenolate mofetil, and rituximab).

**Figure 2:**
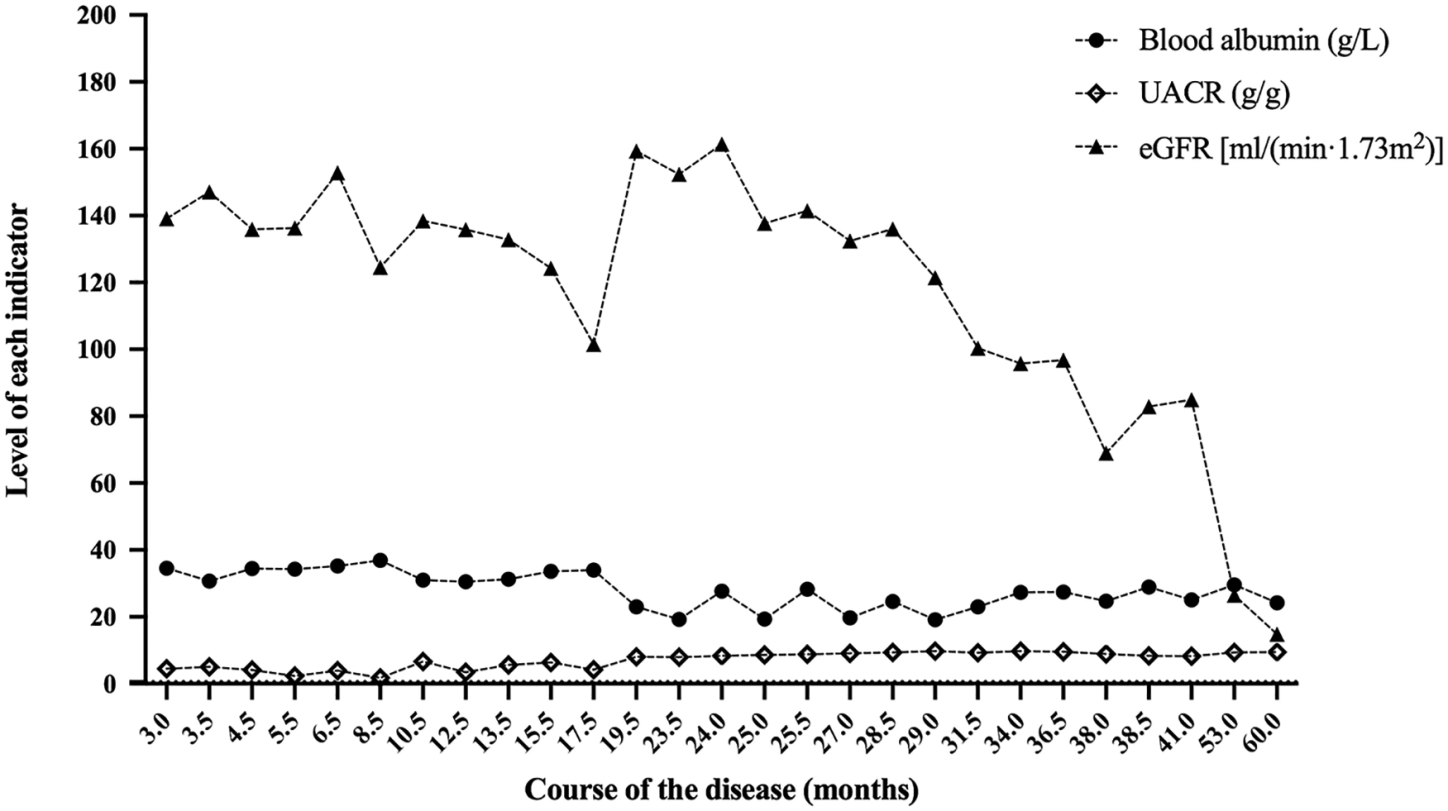
Blood albumin, UACR and eGFR at the follow-up of case 1. After 60.0 months of follow-up, the eGFR of case 1 was 14.8 ml/(min × 1.73 m^2^). UACR, urinary albumin to creatinine ratio; eGFR, estimated glomerular filtration rate.

**Figure 3:**
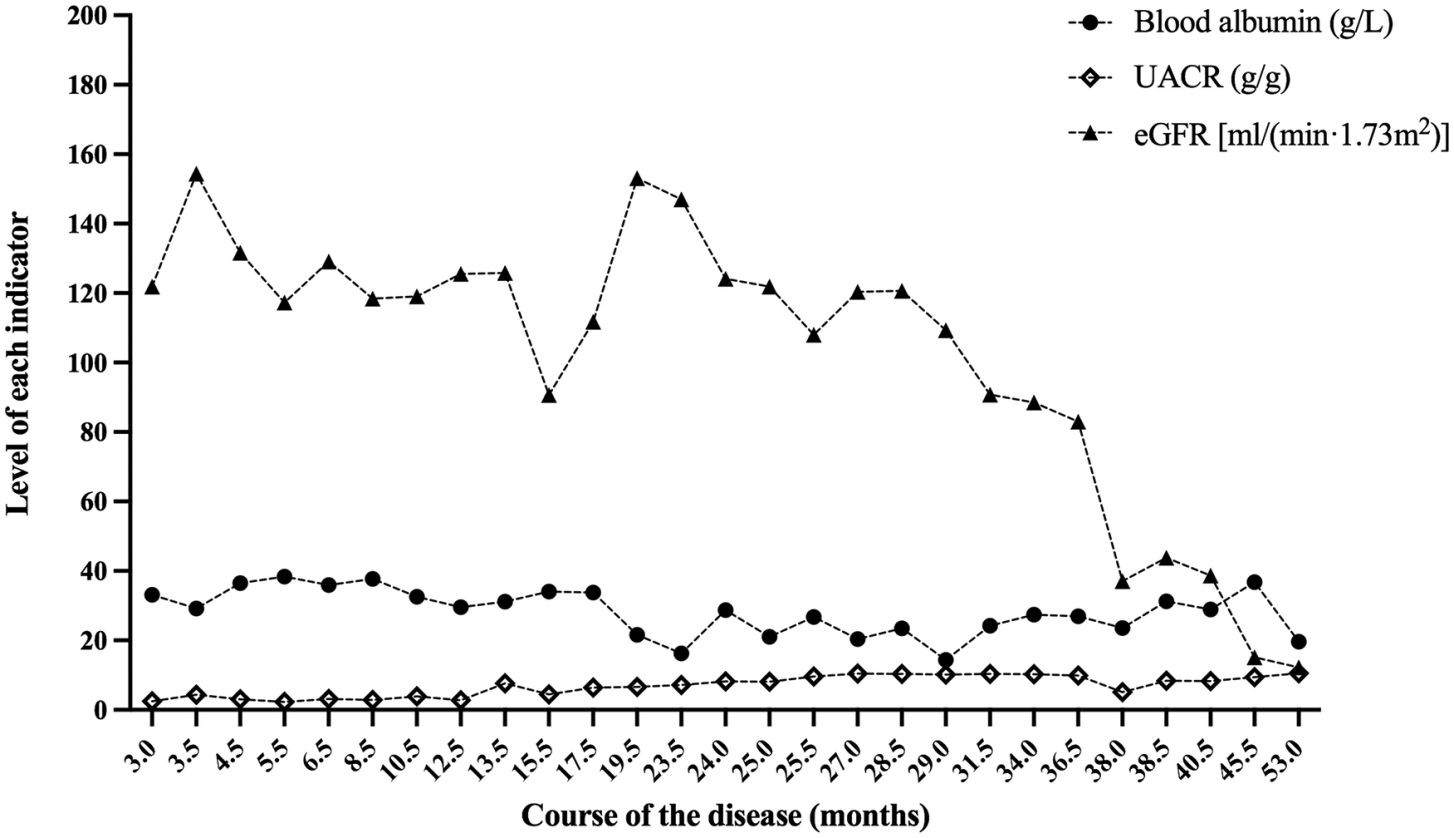
Blood albumin, UACR and eGFR at the follow-up of case 2. After 53.0 months of follow-up, the eGFR of case 2 was 12.2 ml/(min × 1.73 m^2^). UACR, urinary albumin to creatinine ratio; eGFR, estimated glomerular filtration rate.

Combined with the family history of the two children and their 2-year clinical history, we still had a high suspicion that they had genetic SRNS, even though they had a negative genetic testing result. Therefore, a second WES was recommended for this family. The results of the second WES showed a compound heterozygous variant in *SGPL1* in case 1 (Table 1), involving intron4 c.261 + 1G > A (maternal origin, pathogenic variant) and intron12 c.1298 + 6T > C (paternal origin, variants of uncertain significance—likely pathogenic). Sanger confirmed that the patient's sister (case 2) had the same variant, resulting in a compound heterozygous state. Combined with the characteristics of family co-separation, *SGPL1* intronic variant c.1298 + 6T > C, which is classified as PM2 and PP3 by the American College of Medical Genetics and Genomics (ACMG), was judged to be “variant of uncertain significance—likely pathogenic.” Therefore, *SGPL1* was thought to be the responsible gene in monozygotic twins. Later, the two cases were given angiotensin receptor blockers and anticoagulants without glucocorticoids and immunosuppressants. Furthermore, their proteinuria was occasionally slightly reduced. However, they still had nephrotic range proteinuria most of the time, and the proteinuria had not disappeared (Figures 2, 3).

**Table 1:** Whole exome sequencing results of case 1 and case 2.

Case	Gene	Chromosomal position	Mutation information	Zygotic type	Inheritance pattern	Source of variation	ACMG evidence	ACMG variant classification
	* *	Chr10:72610968	NM_003901.4:c.261 + 1G > A			Mother	PVS1, PM2, PM3_Supporting	Pathogenic
Case 1	*SGPL1*			Heterozygous	AR			
		Chr10:72633352	NM_003901.4:c.1298 + 6T > C			Father	PM2, PP3	VUS-LP
		Chr10:72610968	NM_003901.4:c.261 + 1G > A			Mother	PVS1, PM2, PM3_Supporting	Pathogenic
Case 2	*SGPL1*			Heterozygous	AR			
		Chr10:72633352	NM_003901.4:c.1298 + 6T > C			Father	PM2, PP3	VUS-LP

AR, autosomal recessive inheritance; ACMG, American College of Medical Genetics and Genomics; VUS-LP, variants of uncertain significance-likely pathogenic.

The two cases were followed up for 60.0 and 53.0 months, with the last eGFR of 14.8 ml/(min × 1.73 m^2^) and 12.2 ml/(min × 1.73 m^2^), respectively. They all died as a result of abandoning treatment for ESRD. Figures 2, 3, respectively, display the renal function of the two cases during the follow-up. There were no significant extra-renal manifestations at the end of the follow-up (Table 2).

**Table 2:** Extra-renal symptom assessment indicators for case 1 and case 2.

Test	Case 1	Case 2
Blood albumin (g/L)	29.6	36.8
Total cholesterol (mmol/L)	6.78	7.37
Adrenocorticotropic hormone (pg/ml)	39.2	43.9
Serum cortisol (ug/dl)	7.0	8.1
24-hour urine 17-hydroxycorticosteroid quantification (mg/24 h)	0.90	0.94
24-hour urine 17-ketonecorticosteroid quantification (mg/24 h)	1.00	1.10
TSH (uIU/ml)	4.1920	4.4700
T3 (pg/ml)	2.81	2.65
T4 (pg/ml)	0.89	0.88
IgA (g/L)	0.55	0.48
IgG (g/L)	6.60	5.80
IgM (g/L)	0.78	0.67
Complement C3 (g/L)	1.11	0.92
Complement C4 (g/L)	0.41	0.30
Total T lymphocytes (n/ul)	972	1002
Total B lymphocytes (n/ul)	194	205
NK cells (n/ul)	94	91
Ultrasound (adrenal glands)	No abnormalities were seen	No abnormalities were seen
Electrocardiogram	Normal	Normal

### Literature review

We retrieved articles from PubMed, CNKI, and Wan fang databases. Among them, 205 papers were retrieved using the keyword “*SGPL1*[Abstract]*,*” and ten papers were retrieved using the keyword “NPHS14.” One hundred and seventy-three papers remained after the Endnote duplication check. After reading the 173 papers, we selected ten papers according to the inclusion and exclusion criteria. Twenty-nine children with NS caused by *SGPL1* variants reported in ten articles were finally included, and clinical data of two children in our center were analyzed and summarized (Table 3) (4–13).

**Table 3:** Summary of reported cases of nephrotic syndrome caused by *SGPL1* variants (31 cases).

Number	Country or ethnic origin	Age of onset	NS	Age at ESRD or death	Extra-renal phenotype	Chromosome position	Exon	Gene	Protein	Mutation type	Variation classification	Renal biopsy
P101 (6)	Arab	6w	CNS	7w, death	Bilateral adrenal calcification (6w), small penis	Chr10:72636365	14	c.1513C > T	*p*.Arg505*	Homozygous	Pathogenic	/
P102	Arab	2d	CNS	3 m, death	Bilateral adrenal calcification (21w), small penis	Chr10:72636365	14	c.1513C > T	*p*.Arg505*	Homozygous	Pathogenic	FSGS
P103	European	1w	CNS	1 m, ESRD and kidney transplant	Adrenal insufficiency, bilateral adrenal calcification, small penile, cryptorchidism	Chr10:72631618	11	c.934delC	*p*.Leu312Phefs*30	Homozygous	Pathogenic	/
P201 (5)	Türkiye	1m	CNS	5 m, death	Adrenal insufficiency, immunodeficiency, microcephaly, agenesis of the corpus callosum, anemia, failure to thrive	Chr10:72628150	8	c.664C > T	*p*.Arg222Trp	Homozygous	Possibly pathogenic	/
P202	Türkiye	/	CNS	2 m, death	Fetal effusion	Chr10:72628150	8	c.664C > T	*p*.Arg222Trp	Homozygous	Possibly pathogenic	/
P203	Morocco	3m	CNS	6 m, death	Prenatal oedema, ichthyosis (at birth), little calcification of adrenal gland, microcephaly, failure to thrive, hypothyroidism, lymphopenia	Chr10:72631721	11	c.1037G > T	*p*.Ser346lle	Homozygous	Possibly pathogenic	DMS
P204	Morocco	/	CNS	1 m, death	Ichthyosis, microcephaly, deafness, hypotonia, failure to thrive, fetal effusion, lymphopenia	Chr10:72631721	11	c.1037G > T	*p*.Ser346lle	Homozygous	Possibly pathogenic	DMS
P205	Morocco	1m	CNS	3 m, death	Adrenal calcification, microcephaly, epilepsy, lymphopenia, hypothyroidism	Chr10:72631721	11	c.1037G > T	*p*.Ser346lle	Homozygous	Possibly pathogenic	/
P206	Mixed European (Hutterite）	2m	CNS	5 m, ESRD (2 m, peritoneal dialysis)	Adrenal insufficiency (2 m), bilateral sensorineural hearing loss (3y), T and B lymphocyte decrease, anemia, failure to thrive, hypothyroidism (2 m)	Chr10:72633295	12	c.1247A > G	*p*.Tyr416Cys	Homozygous	Possibly pathogenic	FSGS
P207	Spanish	4y	SRNS	Not occurred	Ichthyosis	Chr10:72576615_72576616	2	c.7dup	*p*.Ser3Lysfs*11	Homozygous	Possibly pathogenic	FSGS
P208	Spanish	7m	SRNS	5y, ESRD and kidney transplant; 8y, death	Bilateral adrenal calcification (4 m), adrenal insufficiency (19 m), abnormal gait (15 m), strabismus, cell immunodeficiency (2y)	Chr10:72576615_72576616	2	c.7dup	*p*.Ser3Lysfs*11	Homozygous	Possibly pathogenic	FSGS
P209	Spanish	10m	SRNS	5y, ESRD and kidney transplant; 12y, second kidney transplant; 15y recurrence of massive albuminuria	Ichthyosis (5y), adrenal insufficiency, hypothyroidism,	Chr10:72576615_72576616	2	c.7dup	*p*.Ser3Lysfs*11	Homozygous	Possibly pathogenic	FSGS
P210	France	2y	SRNS	6y, ESRD	Ichthyosis, adrenal insufficiency	Chr10:72614598	5	c.395A > G	*p*.Glu132Gly	Heterozygous	Uncertain significance	FSGS
						Chr10:72630826	10	c.832delA	*p*.Arg278Glyfs*17	Heterozygous	Possibly pathogenic	
P211	France	1y	SRNS	4y, ESRD	Ichthyosis, adrenal insufficiency	Chr10:72614598	5	c.395A > G	*p*.Glu132Gly	Heterozygous	Uncertain significance	FSGS
						Chr10:72630826	10	c.832delA	*p*.Arg278Glyfs*17	Heterozygous	Possibly pathogenic	
P212	Pakistan	19y	SRNS	Not occurred	Adrenal insufficiency	Chr10:72628151	8	c.665G > A	*p*.Arg222Gln	Homozygous	Possibly pathogenic	FSGS
P213	Pakistan	3y	SRNS	7y, ESRD; 8y, kidney transplant	Calcareosis of skin, adrenal insufficiency, failure to thrive, ptosis (25y), paralysis of median and ulnar nerves	Chr10:72628151	8	c.665G > A	*p*.Arg222Gln	Homozygous	Possibly pathogenic	FSGS
P214	Pakistan	2y	SRNS	2.9y, ESRD	Adrenal insufficiency	Chr10:72628151	8	c.665G > A	*p*.Arg222Gln	Homozygous	Possibly pathogenic	FSGS
P215	Mixed European (US)	18y	SRNS	18y, ESRD	Peripheral neuropathy(motor and sensory), multiple mononeuritis, T. B. NK lymphocyte deficiency, hypoglobulinemia, thrombocytopenia, amblyopia, strabismus	Chr10:72619246	7	c.605C > T	*p*.Ser202Leu	Heterozygous	Uncertain significance	FSGS
						Chr10:72631630	11	c.946G > A	*p*.Ala316Thr	Heterozygous	Uncertain significance	
P301 (4)	Pakistan	2.5y	SRNS	5y, ESRD and kidney transplant	Adrenal insufficiency	Chr10:72628151	8	c.665G > A	*p*.Arg222Gln	Homozygous	Possibly pathogenic	FSGS
P302	Türkiye	5.5y	SRNS	5.5y, ESRD and waiting for kidney transplant	Failure to thrive, adrenal insufficiency, mild ataxia, sensorineural deafness, immunodeficiency, ichthyosis, hypothyroidism	Chr10:72637018_72637020	15	c.1633_1635delTTC	*p*.F545del	Homozygous	Possibly pathogenic	FSGS
P303	Peru	0.9y	SRNS	0.9y, ESRD and kidney transplant at the time of report	Adrenal insufficiency, neurological symptoms, Immunodeficiency, cryptorchidism, hypothyroidism, ichthyosis	Chr10:72610968	4 (Intron)	c.261 + 1G > A	*p*.Ser65Argfs*6	Homozygous	Possibly pathogenic	MsPGN
P304	Peru	0.1y	CNS	0.1y, ESRD and peritoneal dialysis	Failure to thrive, adrenal insufficiency, cataract, hypothyroidism, ichthyosis	Chr10:72610968	4 (Intron)	c.261 + 1G > A	*p*.Ser65Argfs*6	Homozygous	Possibly pathogenic	MsPGN
P401 (7)	Saudi Arabia	15y	SRNS	15y, ESRD and hemodialysis	Hemiplegia, right ear deafness	Chr10:72628151	8	c.665G > A	*p*.Arg222Gln	Homozygous	Possibly pathogenic	/
P501 (8)	Pakistan	5m	SRNS	9 m, death	Adrenal insufficiency, failure to thrive, hypothyroidism, bilateral adrenal calcification, persistent lymphocytopenia, ichthyosis, small penis, cryptorchidism	Chr10:72619152	7	c.511A > G	*p*.Asn171Asp	Homozygous	Possibly pathogenic	/
P601 (9)	/	6m	SRNS	29 m, ESRD and peritoneal dialysis; 44 m, death	Adrenal insufficiency, mild expansion of left atrium and left ventricle	Chr10:72631702	11	c.1018C > T	*p*.Arg340Trp	Homozygous	Possibly pathogenic	MsPGN
P701 (12)	/	3m	CNS	16 m, death	Adrenal insufficiency, anemia	Chr10:72631702	11	c.1018C > T	*p*.Arg340Trp	Homozygous	Possibly pathogenic	MsPGN
P801 (10)	/	2m	CNS	3.5 m, ESRD (peritoneal dialysis)	Adrenal insufficiency, hypothyroidism, seizures, severe pulmonary edema	Chr10:72633127	12	c.1079G > T	*p*.Gly360Val	Homozygous	Uncertain significance	MsPGN
P901 (11)	/	6w	CNS	4 m, ESRD	Adrenal insufficiency, hypothyroidism, failure to thrive, immunodeficiency, sensorineural hearing loss, small penile, seizures, choreoid movements	Chr10:72630862	10	c.868T > C	*p*.Phe290Leu	Heterozygous	Possibly pathogenic	MsPGN
						Chr10:72631677	11	c.993C > G	*p*.Tyr331*	Heterozygous	Pathogenic	
P1001 (13)	Afghanistan	1d	CNS	1d, ESRD; 6w, death	Adrenal calcification, cryptorchidism, fetal hydrop, lymphopenia, hypothyroidism, microcephaly, pachygyria and hypoplastic temporal lobes, cerebellar hypoplasia	Chr10:72633281	12	c.1233delC	*p*.Phe411Leufs*56	Homozygous	Pathogenic	/
Case 1	China	5.8y	SRNS	10.5 m, death	Not occurred	Chr10:72610968	4 (Intron)	c.261 + 1G > A	*p*.Ser65Argfs*6	Heterozygous	Pathogenic	IgM nephropathy with glomerulosclerosis and interstitial tubular lesions
						Chr10:72633352	12 (Intron)	c.1298 + 6T > C	/	Heterozygous	VUS-LP	
Case 2	China	5.8y	SRNS	9.8 m, death	Not occurred	Chr10:72610968	4 (Intron)	c.261 + 1G > A	*p*.Ser65Argfs*6	Heterozygous	Pathogenic	/
						Chr10:72633352	12 (Intron)	c.1298 + 6T > C	/	Heterozygous	VUS-LP	

NS, nephrotic syndrome; ESRD, end-stage renal disease; SRNS, steroid-resistant nephrotic syndrome; CNS, congenital nephrotic syndrome; FSGS, focal segmental glomerulosclerosis; DMS, diffuse mesangial sclerosis; MsPGN, mesangial proliferative glomerulonephritis; y, year; m, month; w, week; /, unknown.

VUS-LP, variants of uncertain significance-likely pathogenic. Age of onset, age at first clinical presentation of nephrotic syndrome. End-stage renal disease, the end stage of various chronic kidney diseases that the glomerular filtration rate is less than 15 ml/min/1.73 sqm) and needs to rely on renal replacement therapy to maintain life. Steroid-resistant nephrotic syndrome, persistence of nephrotic range proteinuria following 4 weeks of daily 60 mg/sqm prednisone therapy. Congenital nephrotic syndrome, the presence of nephrotic range proteinuria, hypoalbuminemia, and generalized edema developing within the first 3 months of life.

There were 31 cases in total. The youngest age of onset was one day after birth, and the oldest was 19 years. Among the 31 children, 16 (51.6%) were male, and 15 (48.4%) were female. Genetic testing showed exon variants in 27 (87.1%) cases and intron variants in 4 (12.9%) cases. There were 25 (80.6%) homozygous variants and 6 (19.4%) compound heterozygous variants. In terms of renal phenotype, there were 14 (45.2%) cases of congenital nephrotic syndrome (CNS) and 17 (54.8%) cases of SRNS. Twenty-two cases underwent renal biopsy, and 13 (59.1%) cases had FSGS. Six (27.3%) cases had mesangial proliferative glomerulonephritis (MsPGN), two (9.1%) cases had DMS, and one (4.5%) case had IgM nephropathy. A total of 29 (93.5%) cases had ESRD or were deceased at the time of reporting, with a mean age of occurrence of approximately 34.6 months. Six cases had undergone kidney transplantation, including one case with homozygous frameshift variants in *SGPL1* who underwent re-transplantation, and chronic rejection was seen on graft biopsy.

**Figure 4:**
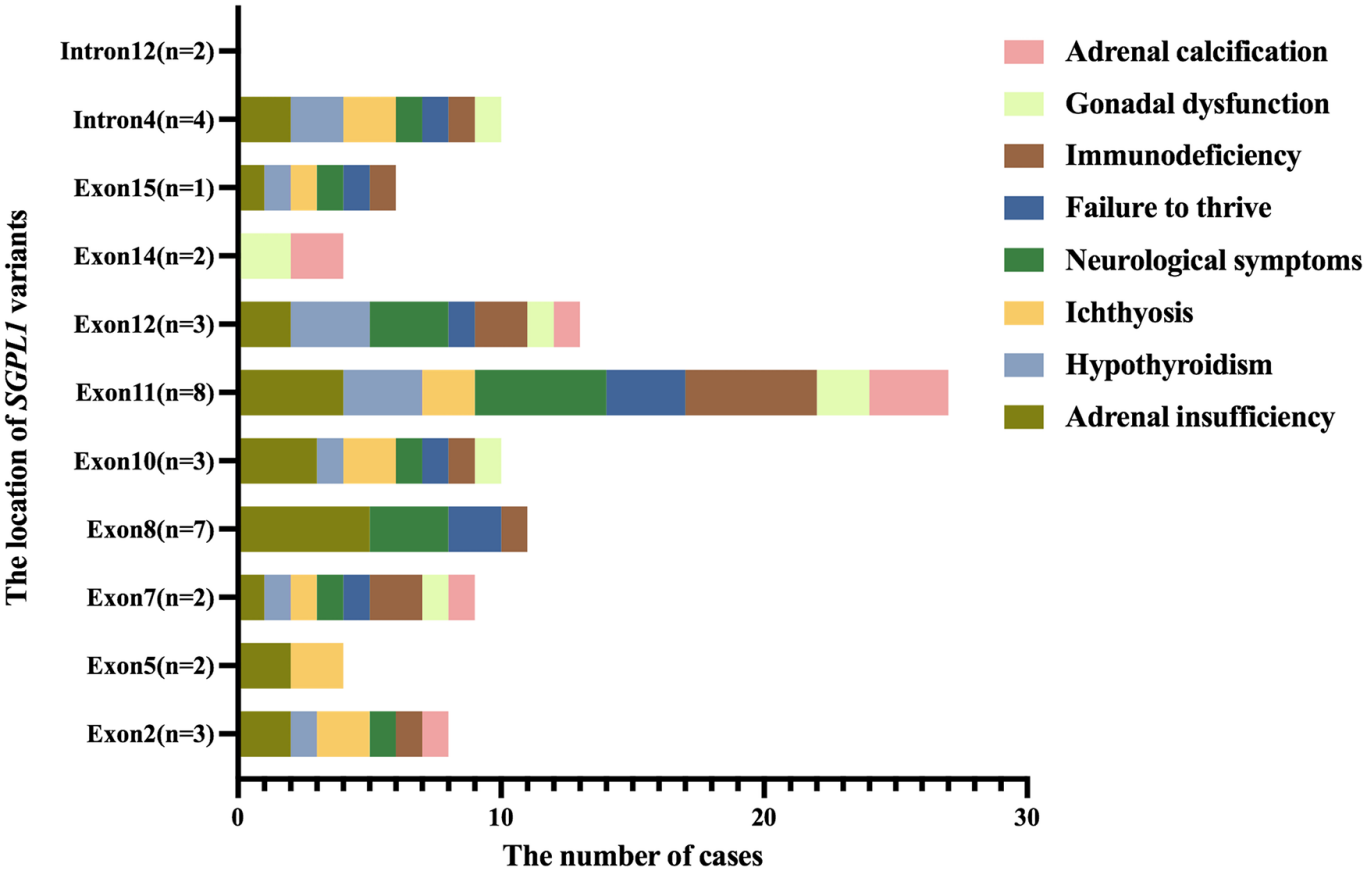
The distribution of extra-renal symptoms at the location of *SGPL1* variants. While intron12 did not show extra-renal symptoms, introns and exons at other sites did, to varying degrees.

All 29 children reported in the literature had extra-renal manifestations, with adrenal insufficiency in 22 (75.9%) cases, neurological symptoms (neurodevelopment delay, hearing loss, hemiplegia, epilepsy, etc.) in 16 (55.2%) cases, immunodeficiency in 14 (48.3%) cases, ichthyosis in 12 (41.4%) cases, hypothyroidism in 12 (41.4%) cases, failure to thrive in 10 (34.5%) cases, adrenal calcification in 5 (17.2%) cases, and gonadal dysfunction in 5 (17.2%) cases. However, two children in our center had no obvious extra-renal manifestations. Additionally, we analyzed extra-renal symptoms by the location of *SGPL1* variants (Figure 4). While intron12 did not show extra-renal symptoms, introns and exons at other sites did, to varying degrees.

### Genotype and clinical phenotype analysis

Among the 31 children with *SGPL1* variants causing NS, fifteen had homozygous missense variants, eight had homozygous frameshift variants, two had homozygous truncation variants, and six had compound heterozygous variants. There were 15 cases of homozygous missense variants, and the age of onset ranged from one month to 19 years (except for two cases whose age at disease onset was unknown), including eight cases of CNS and seven cases of SRNS. A total of 14 of the 15 cases progressed to ESRD or died at the time of reporting, with an average age of about 31.3 months. Regarding renal pathology, there were five cases of FSGS, three cases of MsPGN, and two cases of DMS. There were eight cases of homozygous frameshift variants, and the age of onset ranged from one day to 5.5 years, including three cases of CNS and five cases of SRNS. A total of seven of the eight cases progressed to ESRD or died at the time of reporting, with an average age of about 28.4 months. In terms of renal pathology, there were four cases of FSGS and two cases of MsPGN. There were two cases of homozygous truncation variants, and the age of onset was six weeks and two days, respectively, both of which were CNS. They died in the seventh week and third month of life, respectively. The renal pathology of the case who died in the third month was FSGS. There were six cases of compound heterozygous variants, and the age of onset ranged from 6 weeks to 18 years, including one case of CNS and five cases of SRNS. All six cases progressed to ESRD or died at the time of reporting, with an average age of about 60.1 months. Regarding renal pathology, there were three cases of FSGS, one case of MsPGN, and one case of IgM nephropathy.

## Discussion

In 1990, Richard et al. first described two infants with congenital nephropathy and adrenal calcification, but they could not explain the connection between CNS and adrenal calcification in infants at that time (14). According to two simultaneous reports by Prasad and Lovric et al. in 2017 (4, 5), the relationship between NS and adrenal calcification is caused by *SGPL1* variants. *SGPL1* is located on the long arm of chromosome 10, region 2, band 2, subband 1, contains 15 exons, and encodes SPL, an endoplasmic reticulum enzyme involved in sphingolipid catabolism, catalyzing the cleavage of S1P. SPL guards the only exit point for sphingolipid metabolism, and its inactivation leads to the accumulation of upstream sphingolipid intermediates (15). Moreover, recent studies have shown that SPL deficiency is also closely associated with lung defects, cardiac, urinary tract, renal, and vascular system pathologies, as well as myelodysplasia (16, 17). Experiments have shown that SPL-deficient mice do not survive the first few weeks of life, with an average lifespan of 29 days (16).

Frameshift variants, truncation variants, and missense variants in *SGPL1* are associated with reduced SPL activity or protein deletion (3), causing accumulation of S1P and ceramide, which, in turn, affects cell proliferation, differentiation, and migration. *SGPL1* is widely expressed in human tissues, with higher expression levels in the testis and thyroid and moderate expression levels in the adrenal cortex and kidney. Endothelial cells, mesangial cells, and podocytes in the kidney are typical cells expressing *SGPL1*. An *SGPL1*-deficient mouse model exhibits complete foot process effacement, absence of slit diaphragms (5), and increase in mesangial matrix with obliteration of some capillary lumina (18). We speculate that S1P and ceramide buildup might be responsible for the damage to podocytes and that precise details of these mechanisms merit further investigation. For children with *SGPL1* variants, the pathogenesis of SRNS may be due to excessive S1P that affects the integrity of endothelial cells, the proliferation and migration of mesangial cells, and damage to podocytes.

Numerous studies have shown the crucial role of S1P metabolism in NS and confirmed that defects in S1P metabolism could cause phenotypes seen in other tissues and organs. All 29 children reported in the literature had extra-renal manifestations. Notably, more than 50% of patients had adrenal insufficiency and neurological symptoms (neurodevelopment delay, hearing loss, hemiplegia, epilepsy, etc.). A small number of patients with SRNS also have bone defects, cataracts, amblyopia, strabismus, anemia, and other symptoms. Some patients exhibit an early isolated renal phenotype, which could conceal deficits, such as adrenal insufficiency caused by the initial steroid therapy for nephropathy (19). For *SGPL1* variants, pediatric nephrologists should carefully evaluate the patient's extra-renal symptoms in addition to focusing on the patient's renal phenotype.

Due to the manifestation of steroid resistance, difficult treatment, and prevalence of complications, SRNS remains a challenge for pediatricians. The majority of children with SRNS will develop ESRD and chronic kidney disease. With the development of molecular genetics, more than 50 recessive or dominant genes that can cause SRNS have been identified (20). The two cases described here both displayed steroid resistance at the time of initial treatment, and after genetic testing, SRNS was finally identified as the result of *SGPL1* variants. In children with SRNS with extra-renal manifestations, the possible causative gene should be inferred from the extra-renal manifestations, and the gene should be directly sequenced. Children with CNS and SRNS who present with extra-renal manifestations, particularly adrenal insufficiency, immunodeficiency, developmental delay, and hypothyroidism, should be genetically tested for *SGPL1*. This can rule out NS caused by certain *SGPL1* variants. It is crucial to differentiate genetic or idiopathic forms of SRNS because the two forms of SRNS have very different treatment regimens and prognoses. Genetic SRNS is usually not treated with glucocorticoids and immunosuppressants, whereas those medications are used in idiopathic SRNS. SRNS is the most common acquired cause of ESRD requiring transplantation in children. An Italian study has found that the rate of recurrence after transplantation in idiopathic SRNS was 59.5%, and no relapses occurred in genetic SRNS (21). Their data underline the importance of a genetic evaluation for SRNS genes to plan transplantation, as it represents the principal risk factor for recurrence. If a child can be diagnosed with genetic SRNS through genetic testing at an early stage, a pediatric nephrologist can pay attention to other comorbidities timely and develop the right treatment plan. Besides, they can assess prognosis, such as the risk of recurrence after transplantation, and provide patients and families with the counseling they need.

This paper reported two cases of isolated SRNS caused by *SGPL1* for the first time. Isolated SRNS caused by *SGPL1* is rare. Almost all cases with NS caused by *SGPL1* reported in the literature so far had extra-renal manifestations. However, the two cases were followed up for 60.0 months and 53.0 months, respectively, having no extra-renal manifestations. Unfortunately, they died due to renal failure. Besides, when we initially considered SRNS caused by genetic factors in both children, their genetic testing results did not show any abnormalities. Two years later, we suggested the second WES and found the cause. It was reported in 2017 that *SGPL1* variants can cause abnormal sphingolipid metabolism and are associated with SRNS. After that, the *SGPL1* gene appeared in the updated list of genes associated with SRNS. Thus, the first WES found no pathogenic variants, while the second WES found *SGPL1* variants in both patients in 2018. Our cases highlight the difficulties faced by pediatric nephrologists in the diagnosis and treatment of children with refractory NS. When the initial genetic testing result is negative, the genetic cause cannot be completely ruled out because the Human Gene Mutation Database or ClinVar is constantly being updated.

The limitation of this study must be recognized. The two cases developed ESRD, gave up renal replacement therapy, and died due to renal failure. Thus, our follow-up duration was shorter. If both patients live longer, extra-renal manifestations may occur later.

## Conclusions

In summary, we reported two cases with isolated SRNS caused by compound heterozygous variants in *SGPL1* intron for the first time. By reviewing the related literature, we found that both homozygous and compound heterozygous variants of *SGPL1* almost always had extra-renal manifestations. However, in this paper, the two patients with compound heterozygous variants in *SGPL1* intron had no obvious extra-renal manifestations during follow-up. Moreover, a negative genetic testing result does not completely rule out genetic SRNS because the Human Gene Mutation Database or ClinVar is constantly being updated.

## Data Availability

The original contributions presented in the study are included in the article/Supplementary Material, further inquiries can be directed to the corresponding authors.
